# Forecasting the Largest Expected Earthquake in Canadian Seismogenic Zones

**DOI:** 10.3390/e28020164

**Published:** 2026-01-31

**Authors:** Kanakom Thongmeesang, Robert Shcherbakov

**Affiliations:** 1 Department of Earth Sciences, Western University, London, ON N6A 5B7, Canada; 2 Department of Physics and Astronomy, Western University, London, ON N6A 3K7, Canada

**Keywords:** aftershock events, Canadian seismicity, earthquake clustering, earthquake physics, earthquake simulation, earthquake triggering, ETAS model, seismicity forecasting

## Abstract

Significant earthquakes can cause widespread infrastructure damage, social implications, and substantial economic losses. To mitigate these impacts, earthquake forecasting models have been developed to estimate earthquake occurrences and improve recovery efforts, with the Epidemic-Type Aftershock Sequence (ETAS) model being the most informative statistical framework for characterizing earthquake sequences. In this study, the ETAS model is used to estimate the model parameters for seismicity in Canada using the historical earthquake catalogue and to forecast long-term seismicity for seven different regions in Canada. Furthermore, the model is used to generate synthetic earthquake catalogues in order to assess its ability to replicate observed seismic patterns. The study identifies the southwestern region of Canada, associated with the coastal area of British Columbia, as being at the highest seismic risk, with a 66% exceedance probability for M7.5 events or above to occur in 30 years. In contrast, Alberta features the least seismic risk, with a 4% exceedance probability for events above 6.5 magnitude. For southeastern Canada, associated with Eastern Ontario and Southern Quebec, an exceedance probability of 74% for events above 6.0 magnitude poses the potential for significant damage due to the larger exposed population. Moreover, the resulting seismicity maps show the model’s capability for real-events analysis, but improvements are needed for further applications.

## 1. Introduction

Earthquakes are one of the most unpredictable hazards, being challenging both to analyse and to model [1]. Alongside their intermittent behavior, the hazard intensifies when taking into consideration aftershocks and secondary hazards such as tsunamis, landslides, and liquefaction. The 1976 Tangshan earthquake in China, the 2011 Christchurch earthquake in New Zealand, the 2011 Tohoku earthquake in Japan, and the 2023 Kahramanmaraş earthquake in Turkey are examples of major seismic events that have caused many deaths and billions of dollars in lost assets while leaving a long-lasting impact on those affected [2,3,4,5,6].

To mitigate the societal impact from seismic events, forecasting models have been formulated to estimate earthquake occurrence and increase community preparedness. Earthquake forecasting models can be categorized into three main groups: statistical models, which rely on the record of past seismic events and observed empirical laws; physical models, which utilize the physical triggering mechanism of earthquakes; and hybrid models, which implement both statistical laws and physical information of fractured faults [7,8]. Currently, statistical models and their hybrid variants are considered to be the best-performing models due to their ability to reproduce the number of aftershock [9,10]. Among statistical models, Poisson-based models are widely used due to their simplicity and proficiency in forecasting high-magnitude events [11]. However, these models often disregard impactful aftershock events and underestimate the subsequent damage [12,13,14]. In contrast, the Epidemic-Type Aftershock Sequence (ETAS) model considers the productivity of seismic events and is more capable of capturing the intrinsic characteristics of seismicity compared to Poisson-based models [15,16,17,18,19].

The growing adoption of the ETAS model has made it a standard tool in government agencies and research institutions for real-time operational forecasting and hazard assessment [20,21,22,23]. However, the model’s performance has been observed to vary depending on the target region and completeness of the earthquake catalogue, especially in low- and high-magnitude domains [13,14,24,25,26,27]. Other studies have investigated modifications to the ETAS model, including the incorporation of regional tectonic settings and implementation of a Bayesian framework [9,28,29,30,31]. In [9], the ETAS model was implemented with fault information (ETAS-fault) and the Coulomb failure function (ETAS-cff). These two hybrid variants were evaluated against physical and pure statistical models, which omit seismic empirical laws and rely solely on statistical observations. The results showed that the Short-Term Earthquake Probability (STEP) model, its hybrid STEP-cff variant, and the ETAS hybrid model produced the best-estimated retrospective forecasts for short- and long-term scenarios [9]. Nonetheless, precise tectonic features and stress fields remain difficult to obtain and computationally costly to implement, making hybrid models less favorable. As such, more effort has been dedicated to improving the statistical ETAS model’s accuracy along with its synthetic application.

The studies in [24,32] present some of the more recent works on the ETAS model’s application. In [24], seismic parameters from Central Italy were partially used to calibrate parameters from regions with similar tectonic environments but less data, specifically Türkiye and Croatia. The final analysis revealed the parameters produced for Central Italy to be more reliable and to align well with the observed events, allowing more accurate hazard calculation for Türkiye and Croatia. In concluding, the authors remarked on the model’s dependency on parameterizations and its potential in forecasting future earthquakes. In [32], the authors developed an ETAS forecasting methodology in which the Bayesian method, extreme value statistics, and the ETAS model were used in conjunction to forecast large future aftershocks. The article provided a framework for the ETAS model to analyse the earthquake sequence, allowing the Bayesian predictive distribution to determine the probability of an event exceeding a certain magnitude threshold within a specified time interval. This formulation found success in forecasting probabilities for future large earthquakes in two sequences within the 2016 Kumamoto, Japan earthquake sequence. The study further compared the probabilities derived from the Omori–Utsu law and those from the ETAS model. Ultimately, the Omori–Utsu law overestimated probabilities for extreme events, indicating the need to include subsequent sequences in earthquake analysis and supporting the ETAS model’s effectiveness.

Expanding on the work of [32,33,34], this study intends to address the spatiotemporal ETAS model’s forecasting capabilities and limitations by applying it to seven seismogenic regions in Canada. The Canadian tectonic regions includes intraplate movements, seismically active faults, volcanic zones, the Canadian Cordillera, and the Cascadia subduction zone, the latter being an active convergent margin formed from the Juan de Fuca plate pushing into the North American plate [35,36,37,38]. The different factors that cause strain buildup and influence Canadian seismicity result in vastly different hazardous levels across different territories [35,39]. Accordingly, seven target regions were selected as areas of high seismicity clustering, and the information for Canadian seismicity was obtained from a nationwide earthquake catalogue [40].

Using the catalogue, earthquake events were analysed for completeness and characterized by fitting the ETAS model to obtain the corresponding parameters, then events were synthetically generated for each target region. The process aimed to demonstrate the ETAS model’s performance in approximating regional seismicity, simulating realistic earthquakes, and assessing long-term seismic hazard. First and foremost, the earthquake catalogue was filtered to contain only events in each target region and time. The magnitude–frequency distribution of the events was then plotted against the Gutenberg–Richter relationship at three different cutoff magnitudes to assess the completeness of the extracted events. Afterwards, the ETAS model was fitted to regional seismicity and generated regional seismicity maps, illustrating the background seismicity rate in the area. Lastly, the parameters were used to simulate an ensemble of synthetic earthquake catalogues, from which the highest magnitude events were collected and compiled into an exceedance probability curve. The resulting historical and synthetic seismicity maps served as validating tools for the model’s replication functionality, and the probability curve functioned as a forecasting tool for potential large earthquakes in the future.

As described above, the structure of the paper is as follows: Section 2 covers the details of the earthquake catalogue, the seven target regions, and the formulation of the spatiotemporal ETAS model; Section 3 reports the ETAS model’s outputs, which are the seismicity maps, parameters, and magnitude exceedance probability for each target region; Section 4 discusses the ETAS model’s findings in relation to the regional tectonic setting and factors influencing the model’s results; finally, Section 5 summarizes the ETAS-estimated Canadian seismicity.

## 2. Materials and Methods

### 2.1. Canadian Earthquake Catalogue

For this study, the earthquake catalogue from the National Research Council Canada (NRC) was chosen for its completeness and public accessibility. The NRC records seismic events across the entirety of Canada through the Canadian National Seismograph Network (CNSN), which consists of 165 seismic stations equipped with weak-motion seismometers and strong-motion accelerometers [40].

**Figure 1:**
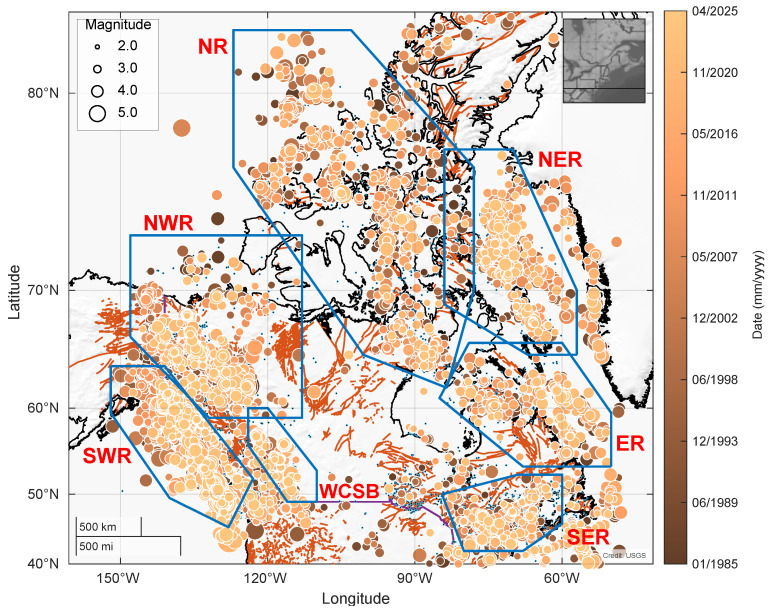
Seismicity map of Canada showing the seven target regions. The solid black and orange lines correspond to the coastlines and fault lines, respectively. The blue polygons encompass the target regions. The seven target regions and their associated territories are: NER (Baffin Bay and nearby islands); NR (Nunavut and the Northwest Territories); ER (Quebec and Newfoundland and Labrador); SER (Eastern Ontario and Southern Quebec); ER (Yukon Territory); SWR (coastal British Columbia); and WCSB (Western Canada Sedimentary Basin). Earthquake events are colored and scaled according to their time of occurrence and magnitude, respectively.

In selecting the seismogenic zones, a seismicity map based on the catalogue was used to assess the spatial distribution of earthquakes. Accordingly, seven prominent Canadian seismogenic zones were outlined, as shown in Figure 1; the blue polygons enclose the seven target seismogenic zones. Similar to previous studies, events occurring outside the polygons and the target time period were included in the seismicity rate calculation but excluded from the parameter estimation process [22].

For each region, the target events were spatially and temporally extracted with cutoff magnitudes of 2.6, 2.8, and 3.0. The three cutoff magnitudes were used to assess the completeness of the filtered regional events, and only events with magnitude 3.0 or higher were adopted for the long-term forecasting. Moreover, the study was restricted to events with a maximum focal depth of 30 km and dated from 1 January 1985 to 5 May 2025, divided into a 10-year training period and a 30-year target period. In this context, the training period was used to estimate the initial seismicity rate based on early events in order to produce a more accurate rate during the target period [32].

### 2.2. The Spatiotemporal ETAS Model

The ETAS model is a hierarchical stochastic point process capable of forecasting the rate of earthquakes that could trigger their own sequences [17,18,19]. The fitting of the model uses earthquake events from a catalogue to describe the seismic behavior through eight parameters. Given a catalogue, the model estimates the background rate and constant seismic parameters associated with each region, in other words “fitting” to the catalogue. Accordingly, the background rate map sheds light on the tectonic activity of each region, while the seismic parameters detail the spatial and temporal behavior of earthquake sequences.

Generally, the model estimates the conditional seismicity rate according to the following equation [22]:

(1) $$\lambda \left(t,x,y,m|H_{t}\right)=\nu \left(x,y\right)+\sum_{i;t_{i}<t}\kappa \left(m_{i}\right)\cdot g\left(t-t_{i}\right)\cdot f\left(x-x_{i},y-y_{i},m_{i}\right),$$

where $\lambda (t,x,y,m|H_{t})$ is the conditional total seismic rate based on the historical earthquake catalogue $H_{t}$ at coordinate (x,y) and time *t*, ν(x,y) is the spatially dependent background rate, $m_{i}$ and $t_{i}$ are the magnitude and occurrence time of event *i*, respectively, while κ(m), g(t), and f(x,y,m) respectively define the mean number, temporal distribution, and spatial distribution of offspring from event *i*. More precisely, the functions κ(m), g(t), and f(x,y,m) can be expressed as follows [22]:

(2) $$\kappa \left(m\right)=A\cdot e^{\alpha (m-m_{c})}\;\mathrm{where}\;m\geq m_{c},$$

(3) $$g\left(t\right)=\frac{p-1}{c}\cdot {\left(1+\frac{t}{c}\right)}^{-p}\;\mathrm{where}\;p\neq 1\;\mathrm{and}\;t>0,$$

(4) $$f\left(x,y,m\right)=\frac{q-1}{\pi \cdot d^{2}\cdot e^{\alpha (m-m_{c})}}\cdot {\left[1+\frac{x^{2}+y^{2}}{d^{2}\cdot e^{\alpha (m-m_{c})}}\right]}^{-q},$$

and

(5) $$f\left(x,y,m\right)=\frac{q-1}{\pi \cdot d^{2}\cdot e^{\gamma (m-m_{c})}}\cdot {\left[1+\frac{x^{2}+y^{2}}{d^{2}\cdot e^{\gamma (m-m_{c})}}\right]}^{-q}.$$

In the above equations, *A*, α, *c*, *p*, *d*, *q*, and γ are the model parameters. It is worth noting that the model has an underlying assumption that the background rate ν(x,y) is composed of a constant and a spatial term v(x,y)=μu(x,y) [22]. Here, μ would be optimized through an iterative scheme that involves the Maximum Likelihood Estimation (MLE) method alongside other model parameters, whereas u(x,y) would be optimized through the declustering process [13,41].

Further, the frequency–magnitude distribution of earthquake magnitudes J(m) is used for the simulation process. The expression of the function J(m) and the Gutenberg–Richter law are respectively provided by [22,42]:

(6) $$J\left(m\right)=\beta \cdot e^{-\beta (m-m_{c})}\;\mathrm{where}\;m\geq m_{c}$$

and

(7) $$\log_{10}N=a-bm,$$

where β is related to the Gutenberg–Richter law’s *b*-value by β=blog10, $m_{c}$ is the cutoff magnitude below which smaller events are not reliably recorded, *N* is the total number of events with magnitude higher than *m*, and *a* and *b* are constant parameters. The parameters *a* and *b* are separately estimated from the distribution of historical earthquake magnitudes, as detailed in [43]. Equation (2) is a form of Utsu’s aftershock productivity law. The equation describes the exponential decrease in the seismicity rate with decreasing earthquake magnitude [44,45]. Similarly, Equation (3) is formulated from the Modified Omori Law (MOL), which describes the decay in seismicity rate with time [46]. Finally, the spatial distribution function *f* may vary from study to study depending on the region of interest. In this instance, Equation (4) corresponds to the ETAS model with seven parameters and Equation (5) corresponds to the ETAS model with eight parameters [22]. For this study, the ETAS model with eight parameters is implemented using Equation (5).

Based on previous works, the parameters θ={ν,A,α,c,d,p,q,γ} are estimated via the MLE method [13,22]. For this study, the MLE framework utilizes the log-likelihood function to determine the most suitable parameters to capture the seismic pattern observed in the historical catalogue. The log-likelihood function is defined as follows [22]:

(8) $$\log L\left(\theta \right)=\sum_{j:\left(t_{j},x_{j},y_{j}\right)\in \left[T_{s},T_{e}\right]\times S}\log\lambda \left(t_{j},x_{j},y_{j}|H_{t_{j}}\right)-\int \int_{S}\int_{T_{s}}^{T_{e}}\lambda \left(t,x,y|H_{t}\right)dtdxdy$$

where logL(θ) represents the log-likelihood score, $\left[T_{s},T_{e}\right]$ denotes the target time interval, *S* denotes the target area, and the subscript *j* denotes the event index. The first term of the equation sums the predicted seismicity rate at the location and time of the historical event *j*. Through Equation (8), the constant parameters are repeatedly revised until the difference between a set of parameters and their previous iteration falls below a threshold, until the best-estimated iteration is produced. Additionally, this framework can be used to evaluate and compare the performance of different models, provided that the models are applied to the same earthquake catalogue.

Another crucial step in parameter estimation is the declustering process. This process uses the background rate *u* and total seismicity rate λ to determine the productivity of an event and probabilistically distinguish independent events from aftershocks. For this purpose, the model compares the background seismicity rate to the rate from every other event at the location of the $i^{th}$ event to determine whether the $i^{th}$ event is induced by the natural seismicity or another event. In this way, the triggering and independent probabilities are calculated for each event. In the same manner as [22,47], after the probabilities are assigned, a random number from a uniform distribution between 0 and 1 is chosen for each event. This randomly generated number is then compared to the background probability of the event. Accordingly, events with a higher background probability than their generated number are deemed to be independent events, and vice versa. Ultimately, the model compiles the independent events to generate a new background rate *u*, repeatedly optimizes this along with the constant parameters θ, then stops when the constant parameters converge.

To summarize, the ETAS model used in this study follows the same estimation process as in [22] and is implemented as reported in [48]. The process refines the model parameters and the background rate through multiple iterations of MLE and declustering processes [22,48]. First, an initial background rate is derived from the historical catalogue and later used as an input to the MLE process defined in Equation (8) to estimate the model parameters. Using the obtained parameters, the first crude iteration of the total seismicity rate is obtained via Equation (1). Next, the total seismicity rate is used to compute the background probabilities for each event, which is used to decluster independent events from aftershocks and generate a new refined background rate. The revised background rate substitutes the initial background rate, and the process repeats itself until the model parameters converge. The final result is an iterated background rate with its associated constant parameters; this dataset best describes the events in the target region. The seismicity maps produced with a 3.0 cutoff magnitude are presented due to the catalogue’s completeness. For reproducibility, the ETAS model implementation reported in this paper and [48] can be downloaded as MATLAB code from the following GitHub link: https://github.com/rshcherb/SMLdecluster, accessed on 1 June 2025.

## 3. Results

### 3.1. Completeness of the Catalogue

The completeness of an earthquake catalogue refers to the assumption that the catalogue contains all events above a certain magnitude threshold. In this instance, the distribution of those events is assumed to follow Gutenberg–Richter scaling [4,25,31,34,49]. As such, deviations from the Gutenberg–Richter scaling in Equation (7) indicate a less complete catalogue. For catalogues with events below the completeness magnitude threshold, the resulting seismicity maps and parameters may not accurately represent the seismic nature of the region. For this purpose, three magnitude cutoffs of 2.6, 2.8, and 3.0 were analysed to determine the completeness of the catalogue for each target region. The magnitude–frequency distribution was then plotted against the estimated Gutenberg–Richter *b*-value associated with each of the three cutoff magnitudes. Thus, the alignment of the magnitude-frequency distribution and the Gutenberg–Richter trend line indicates the cutoff magnitude at which the catalogue is considered complete and suitable for further analysis. For a more comprehensive completeness assessment, ref. [50] progressively increased the cutoff magnitude until at least 90% of events follow the Gutenberg–Richter scaling, providing a standardized method for estimation of the cutoff magnitude.

**Figure 2:**
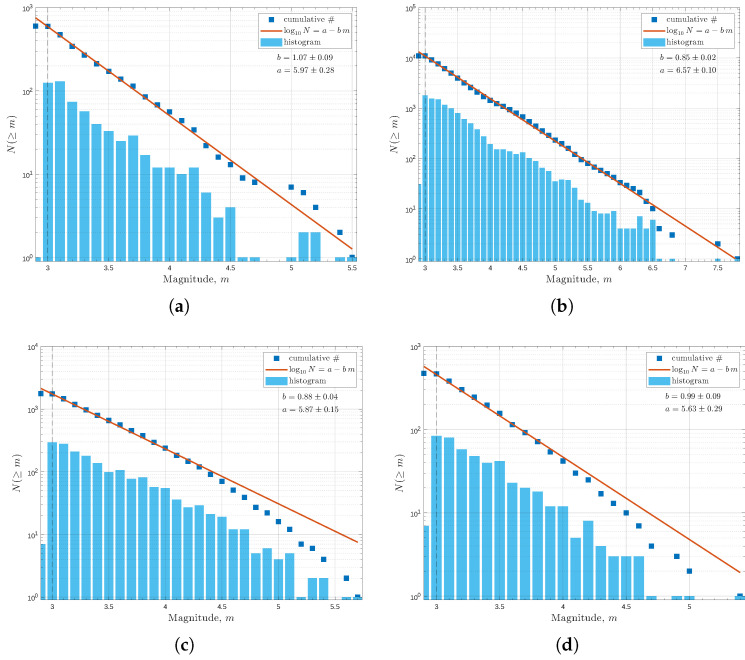
The magnitude–frequency distribution for (**a**) SER, (**b**) SWR, (**c**) NWR, and (**d**) WCSB, with $m_{c}$ = 3.0. The solid blue squares are the cumulative magnitude–frequency distribution of historical events, while the orange line corresponds to the estimated Gutenberg–Richter relationship for cumulative distribution. The histogram in light blue represents the individual magnitude–frequency distribution. The associated Gutenberg–Richter *a*-values and *b*-values are provided in the top right corner of each subfigure. The magnitude-frequency distribution figures for NER, ER, and ER (Figures S1–S3) can be found in the Supplementary Material link listed after the conclusion section.

In Figure 2, the magnitude–frequency distributions for SER, SWR, NWR, and WCSB indicate that a magnitude cutoff value of 3.0 is suitable for ETAS analysis. In general, the cumulative magnitude–frequency distributions (blue squares) align well with the Gutenberg–Richter relationship (orange line) for all target regions, and only deviate slightly near the tail end of the distributions. The observed deviations directly correspond to the number of events in the catalogues, resulting in the event distribution of smaller catalogues deviating more from the Gutenberg–Richter scaling. This is especially the case for NWR and WCSB, which lack high-magnitude events to approximate the tail end of the cumulative plot. With a cutoff magnitude of 3.0, the *b*-values across the seven regions vary from 0.71 ± 0.05 in NR to 1.06 ± 0.09 in SER, while the *a*-values range from 5.00 ± 0.18 in NR to 6.57 ± 0.10 in SWR.

### 3.2. Fitting Regional Seismicity

When fitting the ETAS model, the events used in the estimation were restricted to be inside the target polygons (Figure 1) and within the time interval of 40 years (1 January 1985 to 5 May 2025), and were limited to events with magnitude above 3.0. The first ten years were designated as a preparatory time interval during which the events were used to calibrate the seismicity rate. During the 30-year target time interval, the preliminary parameters of the ETAS model were refined through the process described in Section 2.2 to obtain fitted parameters. The process was repeated for each target region, producing a set of ETAS model parameters for each region.

As shown in Table 1, the ETAS model produces consistent parameter ranges across the seven target regions. The fitted parameters from SER and WCSB show the most variability compared to other regions. From Table 1, SER exhibits unusually low *A* and *d* values, while WCSB exhibits high *A* and *c* values, with a γ value of 0. The γ value of 0 can be interpreted as the region being more suitable for fitting with the version of the ETAS model that has only seven parameters, i.e., Equation (4). The irregular *A* values may reflect an extreme proportion of aftershocks and independent events. In this case, the proportionally greater number of aftershocks in WCSB produces a much higher *A* value than in SER, which has proportionally fewer aftershocks. Furthermore, the low *d* value reflects the clustered nature of events in SER. Finally, the high *c* value in WCSB may indicate a more sustained aftershock sequence; however, further studies are needed to clarify this finding.

**Figure 3:**
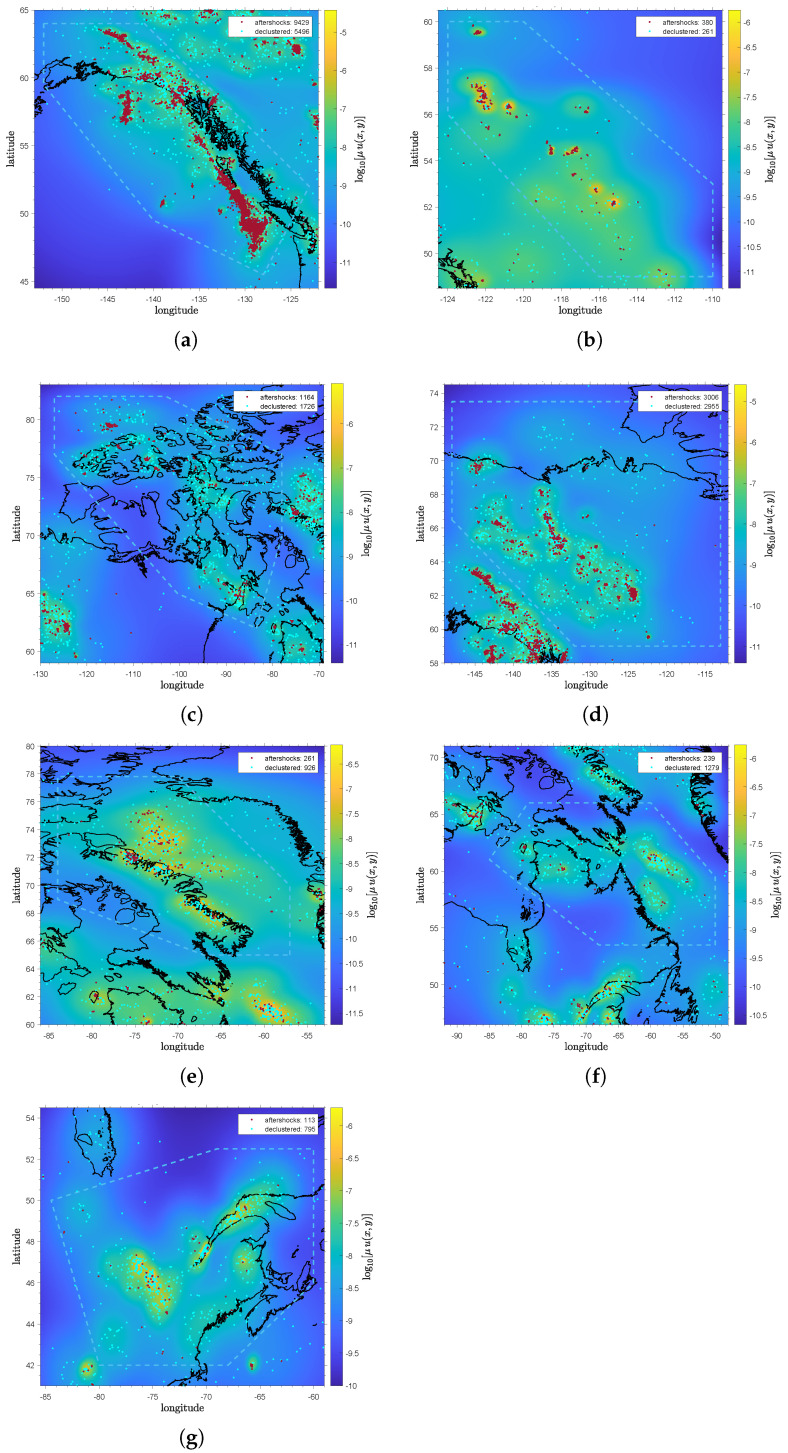
The background seismicity rate maps for (**a**) SWR, (**b**) WCSB, (**c**) NR, (**d**) NWR, (**e**) NER, (**f**) ER, and (**g**) SER. The color bar indicates the logarithmic values of the background seismicity rate ν. The solid black and dashed blue lines outline the coastlines and the target region polygon. Red and blue dots denote the historical aftershocks and declustered/independent events, including those outside of the target polygon.

Among the seismicity maps, the background seismicity rate maps in Figure 3 provide the most informative regional representations. From Figure 3a, the background rate map portrays SWR to be the most seismically active, with 9429 aftershocks out of 14,925 total events. In contrast, WCSB (Figure 3b) exhibits the fewest events, with 380 aftershocks out of 641 total events. Along similar lines, NR and NWR (Figure 3c,d) exhibit low background seismicity rates but a much greater number of events, with 1164 aftershocks from 2890 total events and 3006 aftershocks from 5961 total events, respectively. Conversely, NER (Figure 3e) exhibits the highest background seismicity rates but a lower number of events, with 261 aftershocks from 1187 total events. Lastly, despite being the regions with the highest counts in Canada, ER (Figure 3f) and SER (Figure 3g) exhibit only 239 aftershocks out of 1518 total events and 113 aftershocks out of 908 total events, respectively.

**Figure 4:**
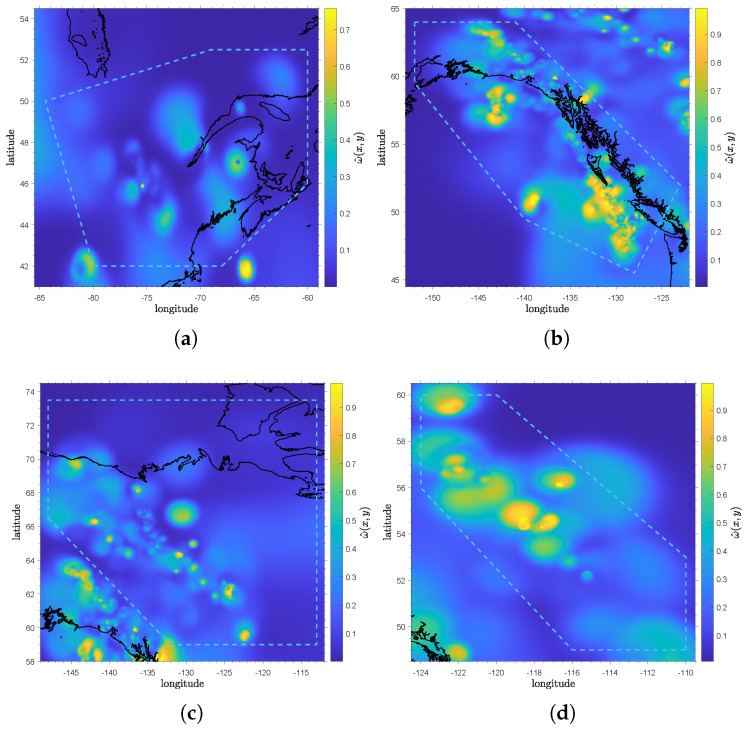
The clustering seismicity rate maps for (**a**) SER, (**b**) SWR, (**c**) NWR, and (**d**) WCSB. The color bar indicates the logarithmic values of the clustering seismicity rate ω. The solid black and dashed blue lines outline the coastlines and the target region polygon. The clustering seismicity rate figures for NER, NR, and ER (Figures S4–S6) can be found in the Supplementary Material link listed after the conclusion section.

As with the background rate map, a clustering rate map can be plotted to depict the triggering rate. Comparing the seismicity maps of SER and SWR highlights the stark difference between the background and clustering rates. In terms of background seismicity, SER features a comparable rate to SWR, as shown in Figure 3e; however, there are fewer aftershocks and independent events compared to SWR. In Figure 4a, the clustering rate of SER is much lower than that of SWR in Figure 4b. This indicates much higher aftershock productivity in SWR despite its relatively comparable background rate. A similar pattern is observed between WCSB and NWR. In Figure 3g and Figure 4c, NWR has a relatively lower background and similar clustering rates but exhibits more events compared to WCSB in Figure 3b and Figure 4d.

**Figure 5:**
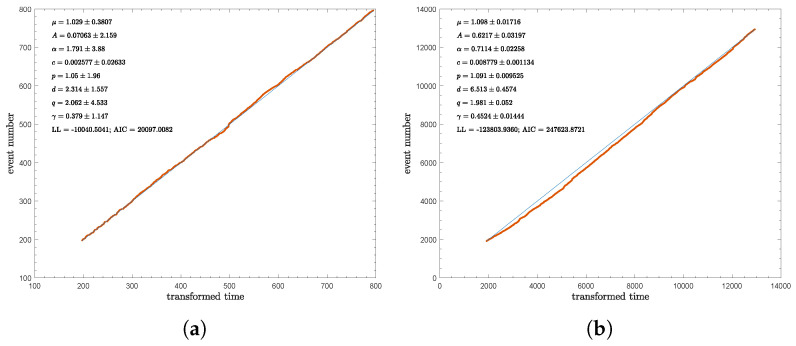

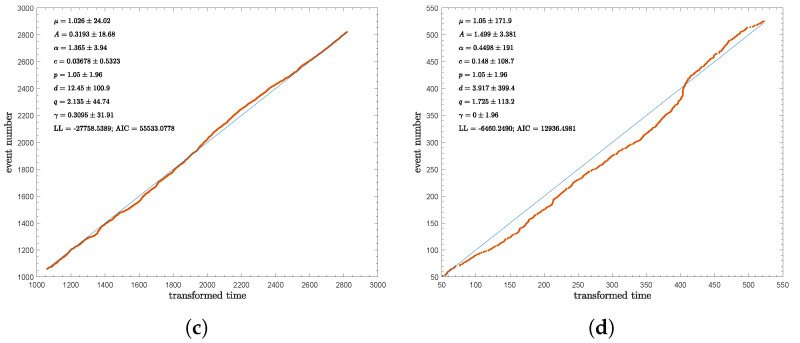
The cumulative event frequency estimated from historical catalogue for (**a**) SER, (**b**) SWR, (**c**) NWR, and (**d**) WCSB. The solid blue and red lines represent the rate at which a given total event is achieved based on the catalogue and the model’s estimation. The transformed time on the x-axis is scaled with the total seismicity rate according to $\tau \left(b\right)=\int_{T_{s}}^{b}\lambda \;dt$.

Lastly, the cumulative event frequency for SER, SWR, NWR, and WCSB can be found in Figure 5. It should be noted that the transformed time was calculated by integrating the total seismicity rate with time; the period represented here corresponds to the target time period. The plots illustrate the cumulative number of events and the corresponding time at which a given total is reached. Using the fitted parameters, the model is capable of recreating the event frequency. The cumulative event frequency observed in the catalogue and based on the model’s estimation demonstrates the model’s performance in depicting seismic frequency. In Figure 5, it can be seen that the model is highly capable of capturing seismic frequency, especially for regions with larger sample sizes. Consequently, the WCSB catalogue produces a suboptimal event frequency estimation due to the lower number of events in this region.

### 3.3. Simulating and Fitting Synthetic Seismicity

In addition to its fitting capabilities, the ETAS model can be used to simulate events and compile synthetic catalogues, which act as different seismic realizations and can be compared to those derived from historical catalogues. Specifically, this study focuses on two simulation aspects: (1) the capability to mimic real seismicity through simulated data, and (2) long-term seismic forecasting through multiple simulations.

For the simulation process, the events generated through the ETAS model were restricted to be inside the target polygons (Figure 1) and with the time interval of 30 years (1 January 1995 to 5 May 2025) while being limited to events with a magnitude above 3.0. After the historical catalogue was fitted, the model utilized the fitted parameters to simulate earthquake events, compile a synthetic earthquake catalogue, and generate synthetic seismicity maps. Assuming that the fitted parameters are suitable seismic representatives, the model would be capable of synthesizing events with comparable magnitude and frequency of occurrence to those in the historical catalogue, thereby providing potential supplemental samples for regions with sparse data.

**Figure 6:**
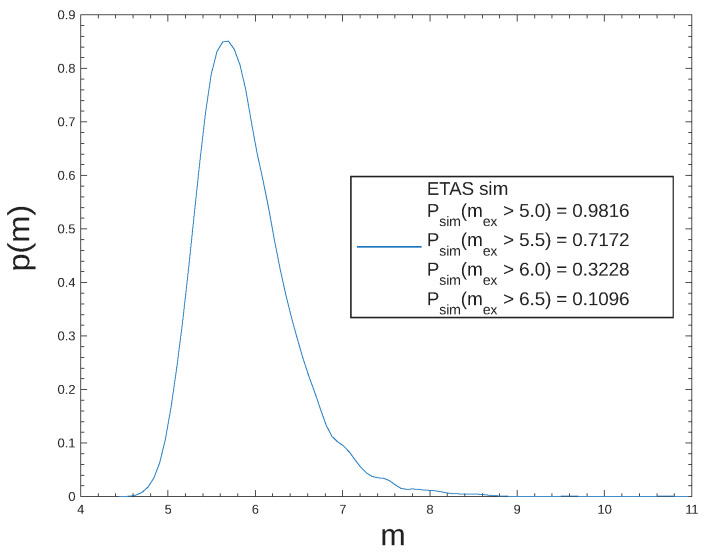
The 30-year probability distribution for the largest expected earthquakes in SER. The blue line represents the probability distribution. A legend for 30-year exceedance probabilities for high-magnitude events is provided.

For forecasting applications, the ETAS model is capable of producing 30-years exceedance probability curves for the largest expected earthquakes through repeated simulations, providing a long-term regional seismicity forecast. For this purpose, the model simulates multiple synthetic catalogues and records the highest-magnitude event that occurred in each catalogue. To elaborate further, the repetition process assumes that multiple copies of synthetic earthquake catalogues are generated, providing multiple seismic realizations for the same time period and spatial region. For this study, we generated 2000 catalogues each for NR, SWR, and WCSB and 5000 catalogues each for the other five regions. In particular, each catalogue contained projected synthetic events for the next 30 years, starting from 2025. As such, the simulations supplied NR, SWR, and WCSB with 2000 highest-magnitude events and the other regions with 5000 events. The extracted maxima were plotted as exceedance probability curves, which represent the probability of a certain magnitude being exceeded within a year, or in this instance within 30 years. An example of the exceedance probability distribution is shown in Figure 6.

The resulting long-term seismic forecasts for Canada indicate a high probability of occurrence for events exceeding magnitude 5.0. As shown in Table 2, SWR, associated with coastal British Columbia, was identified to be at the highest seismic risk, with a 66% exceedance probability for M7.5 events or above to occur in the next 30 years. On the other hand, WCSB was deemed to be the safest, with a 73% exceedance probability for events above 5.0 magnitude and a 0.44% exceedance probability for events above 7.5 magnitude. For ER and SER, primarily associated with Ontario and Quebec, an exceedance probability of 74% and 32% for events above 6.0 magnitude poses the potential for significant damage due to the large exposed population. Moreover, with exceedance probabilities above 40% for events above 6.5 magnitude, NR, NWR, and SWR should anticipate a major earthquake in the upcoming decades.

## 4. Discussion

### 4.1. Correlation to the Canadian Tectonic Background

The seismicity observed in SWR is related to the Cascadia subduction zone near the coast of Vancouver Island, British Columbia, as well as the Denali fault northwest of British Columbia [31,38,39]. The Cascadia subduction zone is an active convergent margin formed from the Juan de Fuca plate pushing into the North American plate [38]. The active margin causes strain buildup, influences volcanic fields, and is most likely responsible for the fault failures and heightened seismicity observed in southern SWR. In [36], the author further highlights Mount Meager and Mount Garibaldi, two seismically active volcano zones in the south of SWR, as being at a very high threat level. A similar conclusion can be found in the Sixth-Generation Seismic Hazard Model of Canada presented in [39], which deemed southwestern British Columbia to be at a high hazard level. In addition, the Denali fault may be related to seismic activity off the northwestern coast of British Columbia [31,51]. From [51], the Denali fault is an active intracontinental fault associated with the colliding Yakutat block. Their collision is capable of influencing the fault systems and triggering strike–slip ruptures, as observed in the 2002 Denali earthquake sequence [25,31,51]. Accordingly, the high seismicity rate observed north of SWR suggests a correlation to the Denali fault; however, further investigation is needed before concluding this hypothesis.

From the seismicity maps of NWR and WCSB, these two regions feature vastly different seismicity rates, suggesting that two different factors dominate regional seismicity. Within NWR, associated with Yukon Territory, the Yakutat terrane is actively colliding with the Pacific margin, resulting in the formation of the northern Canadian Cordillera and high seismicity in southwestern Yukon [37]. This is consistent with our model’s findings, which estimate high clustering rates in the southwestern direction of NWR and a relatively low seismicity rate toward the center. In WCSB, the region features a low seismicity rate and is considered to be the least active among the seven regions. The study in [52] detailed the increase in clustered seismic activity in WCSB using the Nearest-Neighbor Distance (NND) method, the Gutenberg--Richter relation, and the ETAS model. Their study found that this increase is unnatural and most likely to be the result of the recent surge in conventional hydrocarbon production, wastewater fluid injection, and hydraulic fracturing operations. Another study in [53] detailed the seismicity in Alberta from 2011 to 2020 through short-term seismic hazard maps and reached the same conclusion, with the region found to be dominated by induced seismicity. Consequently, the proportional difference in aftershocks and independent events is most likely the result of the fluid pressure change in the terrain due to hydraulic fracturing. As such, the extraction process could increase the Coulomb stress and promote sporadic regional seismic activity observed in WCSB.

In eastern Canada, the three major seismic zones associated with SER (Eastern Ontario and Southern Quebec) are Charlevoix, Western Quebec, and the Lower St. Lawrence. From [35], the observed high seismicity is directly correlated to the reactivation of the Paleozoic rifts due to the compressional stress field driven by the tectonic movement. Among the three zones, Charlevoix is considered to be the most seismically active with the most concentrated event distribution [35,54]. A more recent study in [54] further identified many NE–SW-trending dipping brittle normal faults in the St. Lawrence zone and more E–W-trending faults in the Saguenay graben near Quebec through digital elevation models. Both findings are in agreement with the findings of our ETAS model in terms of the event orientations, and suggest that the brittle faults in the Charlevoix and Western Quebec zones likely contribute to the high background rate and ongoing seismic reactivation in the region. Ultimately, the model successfully identified these three major seismic zones. It additionally identified two more areas with high clustering rates near Miramichi and southern Nova Scotia, most likely related to the northern Appalachian seismic zone [35].

The seismicity observed in the Labrador Sea of ER is the result of intraplate earthquakes on an ancient Mesozoic rift fault, resulting in earthquakes along the continental margin [35]. The ETAS model reveals a consistent conclusion with little to no clustering rate and a NW–SE directional background rate along the continental margin. Lastly, seismicity in NER and NR is not dominated by typical faults per se but by a regional stress field and uplift resulting from postglacial rebound [35,55]. From [55], seismic events near the margin of northern Canada and Greenland, associated with NER, most likely contributed from normal faulting in specific areas due to the ice sheet/glacier movement and deglaciation-induced crustal unloading. The study findings correlate well with NER’s clustering rate map, where the aftershock rates are low but the total number of events is significant. Similarly, the sporadic seismicity rate observed in NR corresponds to areas with thick sediments influencing continental earthquakes, and is not a product of any particular plate boundary activity. Accordingly, further studies should generate and fit multiple seismicity catalogues for each year in order to determine whether deglaciation, which is assumed to progressively increase due to global warming, dominates the contribution to the northern Canada seismicity.

### 4.2. Variability of the ETAS Model

Several factors contribute to the performance of the ETAS model; the completeness of the catalogue, the chosen target area/time, the spatial and temporal kernels, and the parameter bounds are among the factors that can cause variability in fitting and simulation results. This is especially the case for the choice of target polygons, which could underestimate earthquake occurrences due to exclusion of more distant and less impactful outside events. Nonetheless, the results indicate that the ETAS model is capable of fitting Canadian seismicity given sufficient events in the catalogue. This interpretation aligns with previous studies which have confirmed the model’s fitting capabilities and flexibility in case studies of other regions [9,22,23,32]. However, the findings from this study provide additional evidence that the model’s simulation aspect is still in need of further development. In Figure 3, Figure 4 and Figure 5, the model shows the ability to estimate the temporal and spatial behavior of Canadian seismic events; however, the spatial seismicity rate seems slightly too ideal and smoothed. This generalization may be the result of the choice of spatial function *f* or a lack of randomness within the model’s formulation. Regardless, the synthetic catalogue may only be able to partially supplement the historical catalogue rather than providing a full substitute. As such, future work should investigate the spatial function within the standard ETAS model along with the impact of the target area size, and should incorporate a degree of randomness to improve the model’s application to synthetic data.

## 5. Conclusions

This study has found that the ETAS model is able to provide a good fit to Canadian seismicity for seismic events above a 3.0 cutoff magnitude. The model estimation shows that Baffin Bay and the nearby islands feature the highest background rate and that British Columbia produces the most aftershocks, with 9429 aftershocks out of a total of 14,925 events in the past 30 years. Moreover, the model finds the coastal area of British Columbia to be at the highest seismic risk, with a 66% exceedance probability for events above 7.5 magnitude to occur in the next 30 years. On the other hand, Alberta is deemed the safest, with a 4% exceedance probability for events above 6.5 magnitude. The model was further evaluated for its simulation performance, with synthetic events found to be well-generated temporally but not spatially. In conclusion, British Columbia, Eastern Ontario, and Southern Quebec are at risk for major seismic hazards due to the relatively high seismicity rate and exposed population. Moreover, despite the small number of recorded events, Alberta should be cautious of anthropogenic energy-related operations due to the associated seismic risks.

## Figures and Tables

**Table 1:** Estimated ETAS parameters for the seven regions using the Canadian earthquake catalogue with events above magnitude cutoff $m_{c}=3.0$.

Region	NER	NR	ER	SER	NWR	SWR	WCSB
μ	1.14	1.02	1.09	1.03	1.03	1.10	1.05
*A*	0.20	0.12	0.13	0.071	0.32	0.62	1.50
α	1.30	1.42	1.18	1.79	1.36	0.71	0.45
*c*	0.015	0.012	0.0039	0.0026	0.037	0.0088	0.15
*p*	1.05	1.11	1.07	1.05	1.05	1.09	1.05
*d*	10.42	19.37	8.02	2.31	12.45	6.51	3.92
*q*	1.68	1.83	2.17	2.06	2.14	1.98	1.72
γ	0.60	0.37	0.75	0.38	0.31	0.45	0.00
*b*	0.95	0.72	0.82	1.07	0.88	0.85	0.99
$N_{target}$	622	709	406	599	1761	11,015	474

**Table 2:** Probabilities of the largest expected earthquakes in the next 30 years for seven Canadian seismogenic zones.

Region	P$\left(m_{\mathrm{ex}}>5.0\right)$	P$\left(m_{\mathrm{ex}}>5.5\right)$	P$\left(m_{\mathrm{ex}}>6.0\right)$	P$\left(m_{\mathrm{ex}}>6.5\right)$	P$\left(m_{\mathrm{ex}}>7.0\right)$	P$\left(m_{\mathrm{ex}}>7.5\right)$
NER	99.8	89.8	54.6	23.9	9.0	3.0
NR	100.0	99.5	93.6	71.8	43.4	20.6
ER	100.0	96.4	73.7	42.3	21.0	9.4
SER	98.2	71.7	32.3	11.0	3.5	1.1
NWR	100.0	100.0	95.9	73.9	40.8	16.9
SWR	100.0	100.0	100.0	99.3	90.3	66.5
WCSB	73.0	34.4	13.2	4.5	1.5	0.4

## Data Availability

The Canadian earthquake catalogue was downloaded from https://www.earthquakescanada.nrcan.gc.ca/stndon/NEDB-BNDS/bulletin-en.php, accessed on 1 June 2025.

## Supplementary Materials

The following supporting information can be downloaded at: https://www.mdpi.com/article/10.3390/e28020164/s1, Figure S1: The magnitude-frequency distribution for NER with $m_{c}$ = 3.0; Figure S2: The magnitude-frequency distribution for NR with $m_{c}$ = 3.0; Figure S3: The magnitude-frequency distribution for ER with $m_{c}$ = 3.0; Figure S4: The clustering seismicity rate maps for NER; Figure S5: The clustering seismicity rate maps for NR; Figure S6: The clustering seismicity rate maps for ER.
