# Supplementary material for: Forecasting the Largest Expected Earthquake in Canadian Seismogenic Zones
Source: Entropy (Basel). 2026 Jan 31;28(2):164. doi: 10.3390/e28020164 (PMC12939165; doi:10.3390/e28020164)
Supplement: Supplementary file 1 [file entropy-28-00164-s001.zip › entropy-4106209-supplementary.pdf]

Supplemental Material for:

Forecasting the largest expected earthquake in Canadian seismogenic zones

Kanakom Thongmeesang[1‡] and Robert Shcherbakov[1,2‡]

[1]Department of Earth Sciences, Western University,
London, Ontario, *N6A 5B7*, Canada
[2]Department of Physics and Astronomy, Western University,
London, Ontario, *N6A 3K7*, Canada

[‡]E-mail: kthongme@uwo.ca, rshcherb@uwo.ca

January 29, 2026

S1 Supplementary Figures

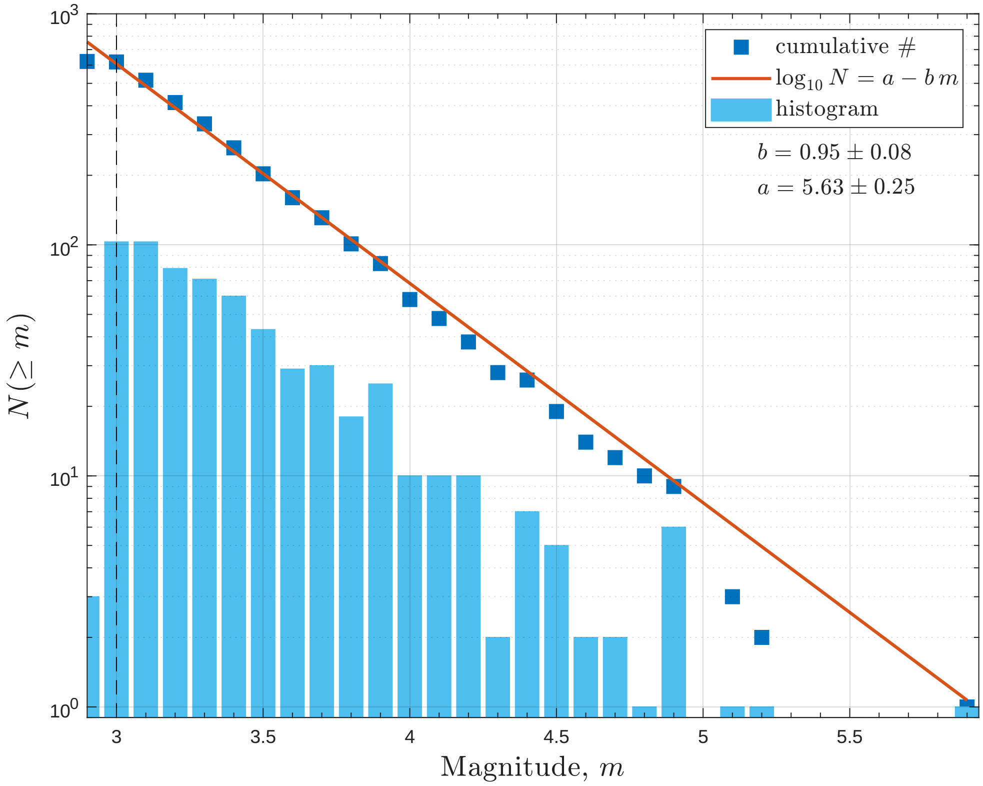

Figure S1: The magnitude-frequency distribution for NER with $m_c = 3.0$. The solid blue squares are the cumulative magnitude-frequency distribution of historical events. While the orange line corresponds to the estimated Gutenberg-Richter relationship for cumulative distribution. The histogram in light blue represents the individual magnitude-frequency distribution. The associated Gutenberg-Richter a- and b-values are provided in the top right corner of each subfigure.

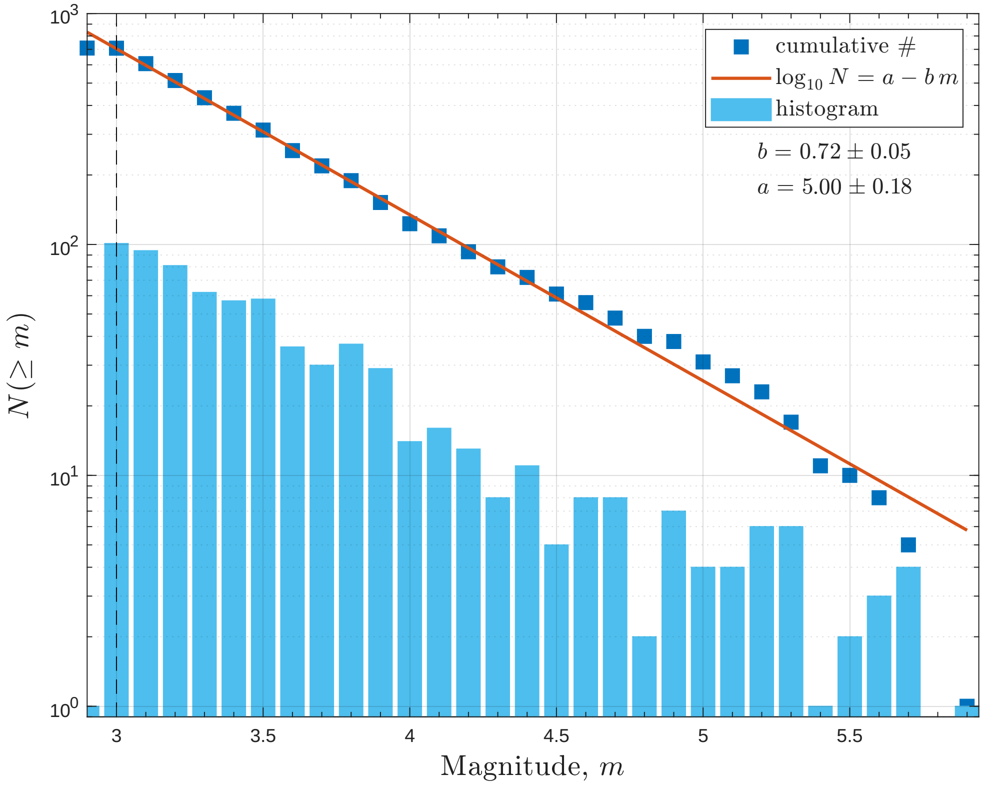

Figure S2: The magnitude-frequency distribution for NR with $m_c = 3.0$. The solid blue squares are the cumulative magnitude-frequency distribution of historical events. While the orange line corresponds to the estimated Gutenberg-Richter relationship for cumulative distribution. The histogram in light blue represents the individual magnitude-frequency distribution. The associated Gutenberg-Richter a- and b-values are provided in the top right corner of each subfigure.

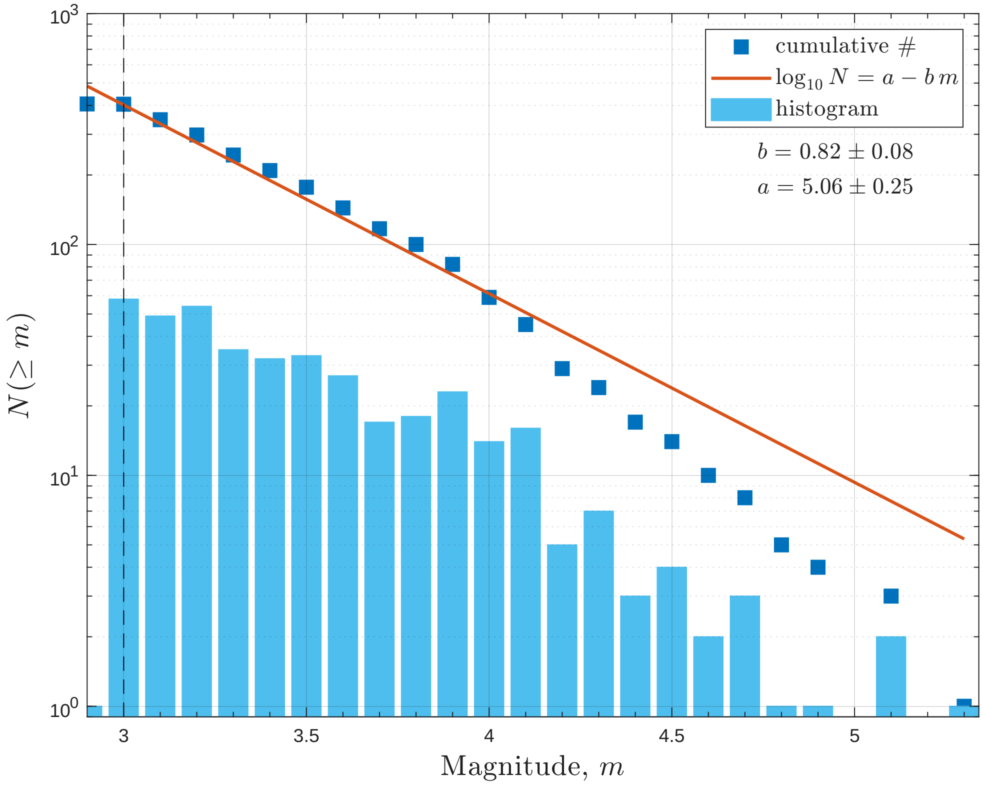

Figure S3: The magnitude-frequency distribution for ER with $m_c = 3.0$. The solid blue squares are the cumulative magnitude-frequency distribution of historical events. While the orange line corresponds to the estimated Gutenberg-Richter relationship for cumulative distribution. The histogram in light blue represents the individual magnitude-frequency distribution. The associated Gutenberg-Richter a- and b-values are provided in the top right corner of each subfigure.

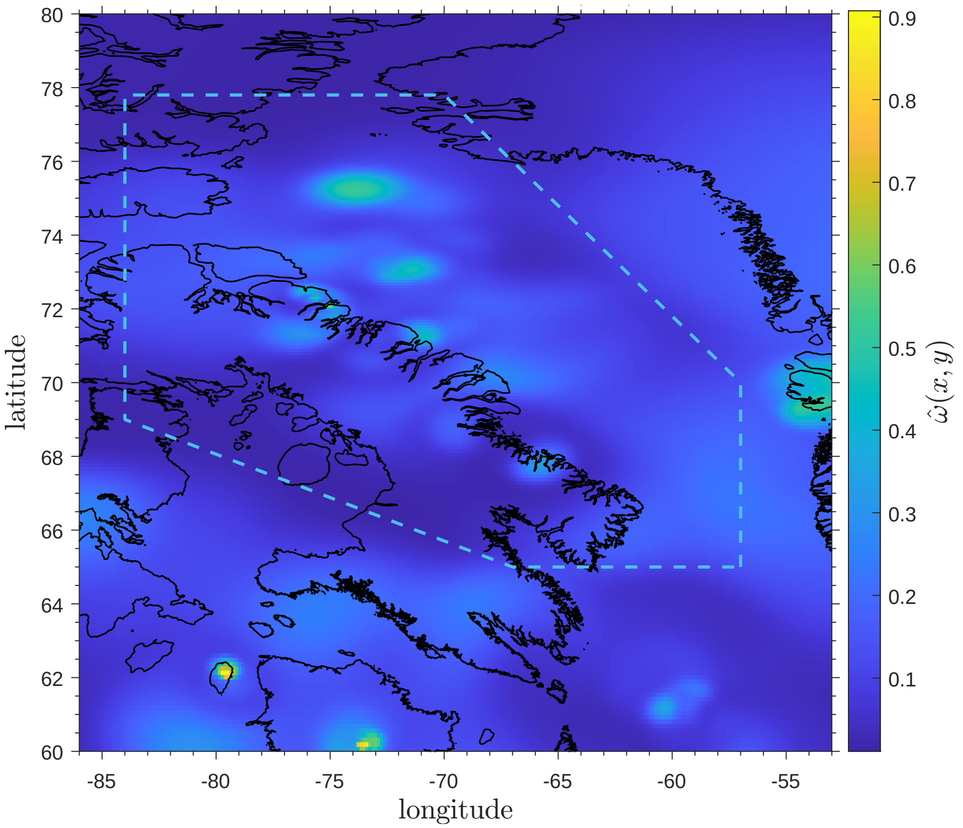

Figure S4: The clustering seismicity rate maps for NER. The color bar indicates the logarithmic values of the clustering seismicity rate, ω. The solid black and dashed blue lines outline the coastlines and the target region polygon.

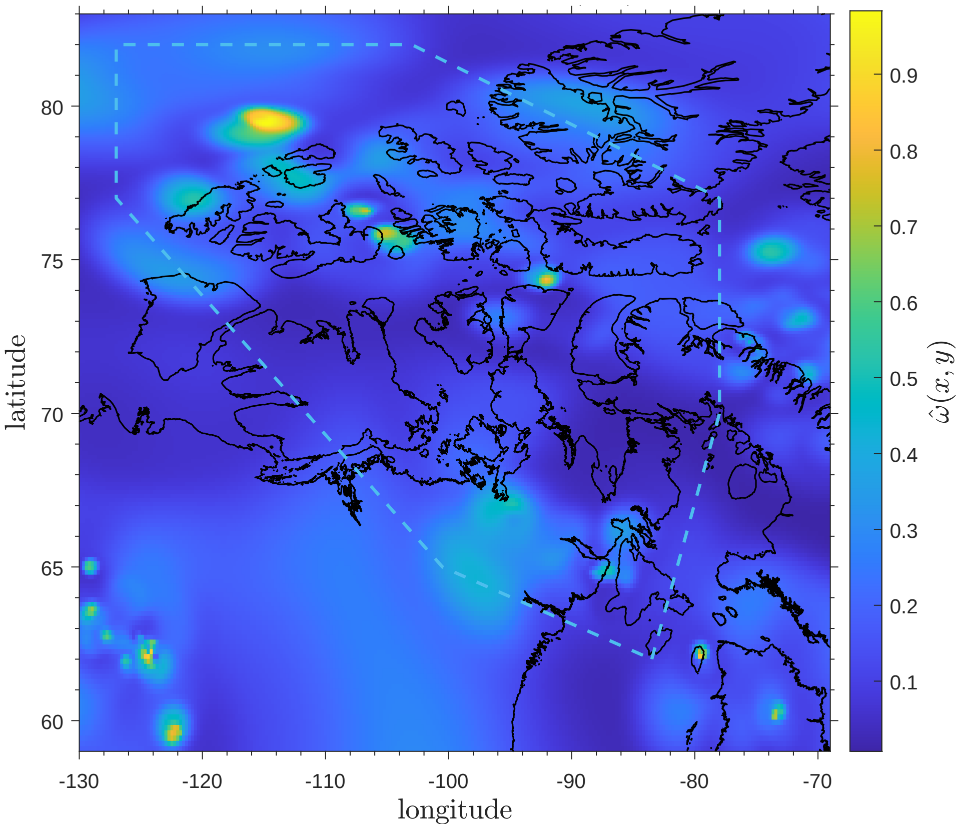

Figure S5: The clustering seismicity rate maps for NR. The color bar indicates the logarithmic values of the clustering seismicity rate, ω. The solid black and dashed blue lines outline the coastlines and the target region polygon.

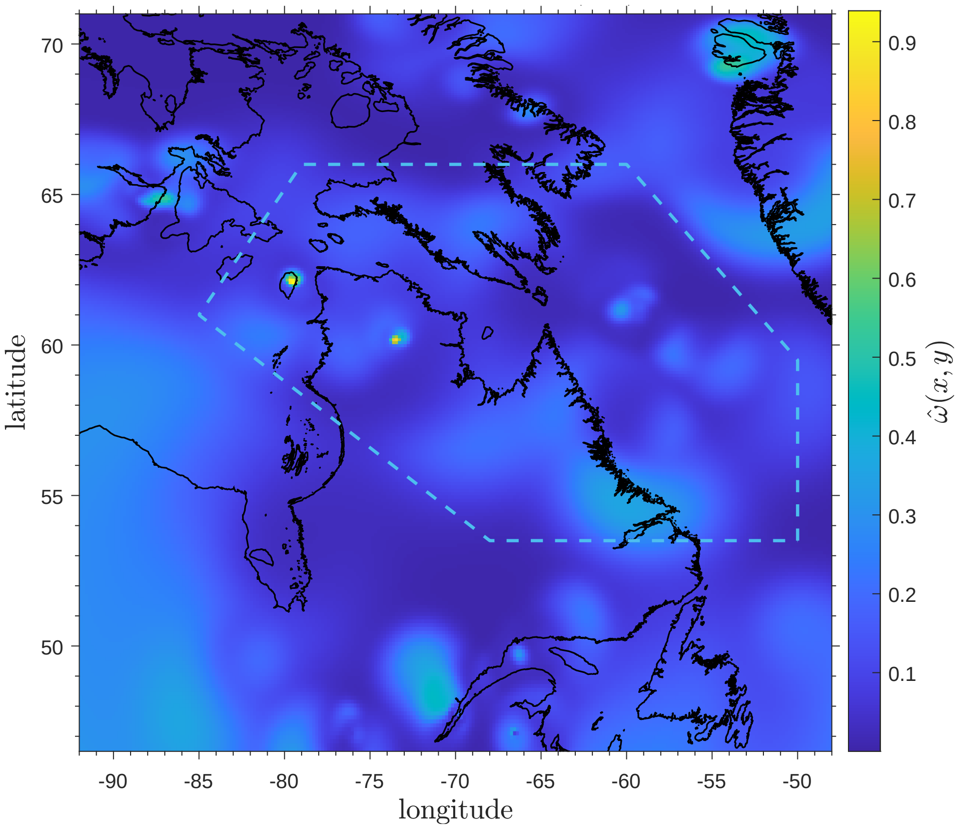

Figure S6: The clustering seismicity rate maps for ER. The color bar indicates the logarithmic values of the clustering seismicity rate, ω. The solid black and dashed blue lines outline the coastlines and the target region polygon.
